# Orthotopic Heart Transplantation for Congenital Heart Disease with Dextrocardia: A Single-Center Clinic Experience

**DOI:** 10.1155/2020/3487635

**Published:** 2020-05-31

**Authors:** Guohua Wang, Yixuan Wang, Jing Zhang, Yongfeng Sun, Jie Cai, Jinping Liu, Nianguo Dong

**Affiliations:** Cardiovascular Surgery, Wuhan Union Hospital, Huazhong University of Science and Technology, Wuhan, China

## Abstract

**Background:**

We report a modified transplantation surgical technique for CHD with dextrocardia which is rare and surgically challenging.

**Methods:**

From January 2015 to May 2018, 5 patients with end-stage CHD with dextrocardia underwent heart transplantation at our institute. They were 10, 29, 13, 15, and 22 years old, respectively; 3 of them had dextroversion, and the other 2 had mirror-image dextrocardia and post-TCPC. The atrial-atrial anastomosis was performed first between the donor's upper-left PVO and the recipient's lower-left PVO. The apex thereby rotated approximately 90° clockwise (to the right). The end-to-end donor and recipient aortas, vena cava, and pulmonary arteries were then anastomosed.

**Results:**

The cold ischemic time of the donor heart was 284.6 ± 108.3 min, and the CPB time was 190.2 ± 43.8 min. The postoperative X-ray showed the apex on the right. Four patients were successfully discharged, and the follow-up times were 47 months, 36 months, 12 months, and 12 months. One post-TCPC patient died because of pneumonia and hypoxia at 59 postoperative days.

**Conclusions:**

Heart transplantation with dextrocardial CHD is rare. A 90° rotation at the left atrial level, aortic end-to-end anastomosis, and vena cava reconstruction by vascular prosthesis or systemic atrial cuff is a simple and effective surgical strategy.

## 1. Introduction

Since Barnard performed the first heart transplant operation in 1967 [1], heart transplantation has gradually become an effective treatment for patients with end-stage heart disease. The data of the International Society of Heart and Lung Transplantation (ISHLT) show that approximately 5,000 heart transplantations are performed worldwide every year, but heart transplantation for CHD comprises only 3%-4% of those procedures [2]. In China, heart transplantation has grown vigorously in the last ten years after donor after brain death (DBD) started to be widely implemented. More than 450 patients per year have undergone heart transplantation in China, but recipients with CHD make up less than 50 such cases, which is consistent with ISHLT data. Heart transplantation for CHD is technically more difficult than for non-CHD patients, and children's CHD heart transplantation has been limited by the relatively small number of matched donors. Dextrocardia, including dextroversion (situs solitus or ambiguous) and mirror-image dextrocardia (situs inversus totalis), is a rare malformation found in 1 in 12,000 people [3]. Moreover, since it is hard to find a dextrocardia donor, heart transplantation for patients with dextrocardial CHD is more challenging.

From January 2015 to May 2018, 5 heart transplantations into patients with dextrocardial CHD were successfully completed at our institute. The normal donor hearts (S.D.S.) were implanted into recipients with dextrocardia and complex CHD (listed in Table 1). Through this report, we contribute our experience managing a unique transplant with a focus on the surgical techniques employed.

## 2. Materials and Methods

All donor grafts were donated to the Red Cross Society of Hubei Province and were allocated by the China Organ Transplant Response System. The study was approved by the Ethics Committee of Tongji Medical College of Huazhong University of Science and Technology (IORG No: IORG0003571) and was performed in accordance with the national program for deceased-donor organ donation in China [4] (national protocol for China category I [5]). The clinical and research activities being reported are consistent with the principles of the Declaration of Istanbul and Declaration of Helsinki. Written informed consent was obtained from all patients or their guardians.

### 2.1. Study Population

Heart transplantation patients in the Cardiovascular Surgery Department, Wuhan Union Hospital of Tongji Medical College, from January 2015 to May 2018 were eligible. Among them, 5 heart transplant patients were diagnosed with dextrocardia and complex CHD. Medical records were retrieved from the cardiovascular transplant database in our center. The indication for heart transplantation for older children and adults was based on advanced New York Heart Association (NYHA) class III/IV, cardiopulmonary test results, and hemodynamic data. Decompensated cyanosis and protein-losing enteropathy in patients with the Fontan procedure were also considered as indications for heart transplantation.

### 2.2. Organ Preservation and Operative Technique

Donor cardiectomy was accomplished with en bloc removal of the superior vena cava (SVC) and extra length on the inferior vena cava (IVC) with the innominate vein, aorta, and pulmonary artery. The donor heart was then prepared by marking the upper-left and lower-right pulmonary vein orifices and then trimming the left atrium. A uniform method of preservation was applied to all donor hearts and consisted of 1 l of cold (4°C) histidine-tryptophan-ketoglutarate (HTK) solution during transport. Additionally, 500 ml of HTK solution was perfused before implantation.

### 2.3. Immunosuppressive Therapy

Interleukin-2 receptor antagonist (IL-2RA, basiliximab) and methylprednisolone were used for induction therapy. The first dose of basiliximab (20 mg for children < 35 kg, 40 mg for children ≥ 35 kg) was administered intravenously 2 h prior to the operation. The second dose was administered intravenously 4 days after transplantation. The first dose of methylprednisolone (500 mg) was added into the priming solution of the cardiopulmonary bypass machine, and the second dose (500 mg) was administered intravenously before aortic cross-clamp removal. Upon return to the ICU, patients were administered 4 125 mg doses of methylprednisolone q8h. Subsequent doses were administered q8h, and each dose was reduced by 25 mg until methylprednisolone was discontinued. Then, methylprednisolone (1 mg/kg) was orally taken twice per day. The dose of methylprednisolone was gradually reduced over one month, and the final maintenance dose was 10 mg qd (0.2 mg/kg for children). Maintenance immunotherapy consisted of tacrolimus (0-90 days: 10 ng/ml; 90 days-1 year: 8-10 ng/ml; >1 year: 5-8 ng/ml), mycophenolate (maintenance dose 1.5-2 g/d for adults, 600 mg/m^2^/d for children), and prednisone which were used for all recipients. Patients who had vascular prosthesis for vessel reconstruction were administered aspirin 3-5 mg/kg/d for children and 100 mg/d for adults.

### 2.4. Outcome Measures

Demographic and clinical characteristics of all heart transplant donors and recipients were examined. After being discharged from our hospital, all patients were admitted to the outpatient department weekly for the 1st month, biweekly until the 3rd month, monthly from the 4th to the 12th month, and twice for one year thereafter. May 1st, 2019 was set as the end point of this study. Patients were evaluated for heart function and quality of life, biochemical tests (including fasting glucose, liver and renal function, blood concentration of tacrolimus, CMV antibody titers, and serous myocardial enzyme), echocardiography, electrocardiography, and chest X-ray. Heart biopsies were performed when graft rejection was suspected based on the serous myocardial enzyme, echocardiography, and electrocardiography.

### 2.5. Statistical Analysis

Unless otherwise stated, continuous variables conforming to a normal distribution are expressed as the mean ± standard deviation. Variables fitting a skewed distribution are reported as the median (interquartile range (IQR)). Survival rates were calculated using Kaplan-Meier's curves. All statistical calculations were performed with SPSS 23.0 for Windows (SPSS, Chicago, IL, USA).

## 3. Results

### 3.1. Donor Characteristics

Five normal (S.D.S.) donor hearts were voluntarily contributed by brain-dead patients. Three male donors were blood type B, 1 female donor was blood type O, and 1 male donor was blood type O. The mean age and weight were 31.2 ± 14.3 years old and 54.0 ± 9.6 kg, respectively. The mean cold ischemic time was 284.6 ± 108.3 min. The causes of brain death were cerebrovascular accident, cerebral tumor, and cerebral trauma. The donor characteristics are shown in Table 1.

### 3.2. Recipient Characteristics

Three patients were male. The mean age and weight at heart transplantation were 18.0 ± 7.4 years old and 45.8 ± 18.5 kg, respectively. Four patients were blood type B, and one patient was blood type O. The mean PAP, preoperative LVEF, and BNP were 24.8 ± 10.2 mmHg, 33.8 ± 17.6%, and 3739.8 ± 1044.3 pg/ml, respectively. The NYHA classes were IV, III, IV, IV, and IV, respectively. The recipient characteristics are shown in Table 1 and Table 2.

For the congenital malformation, there are 3 letters in which the first one stands for visceroatrial situs: S for situs solitus, I for situs inversus, and A for situs ambiguous; the second one stands for ventricular loop: D for solitus ventricles, L for L-loop, and X for X-loop; and the third one stands for conotruncus: S for situs solitus, I for situs inversus, and D, L, and A for the other positions of the aorta and pulmonary artery.

The primary diagnosis of patient #1 was dextroversion (A.X.L.), situs solitus, right atrial isomerism, single atrium, single ventricle, common atrioventricular canal (type C), severe common atrioventricular valve insufficiency, pulmonary stenosis, and noncompaction of ventricular myocardium. The patient had marked cyanosis of her extremities and face, palpitations and dyspnea on exertion, and recurrent edema of the lower limbs for approximately 4 years, and the above clinical symptoms became severe during the 2 years preceding surgery.

The primary diagnosis of patient #2 was dextroversion of the heart (S.D.S.), situs solitus, double-outlet right ventricle, ventricular septal defect, bilateral superior vena cava, pulmonary hypertension, noncompaction of the left ventricular myocardium, mild or moderate mitral regurgitation, and arrhythmia. The patient was hospitalized in our hospital due to recurrent cough, expectoration, palpitations and dyspnea for 2 months, and worsening for approximately 10 days. Medical treatment did not resolve clinical symptoms, and the patient had to be resuscitated with CPR due to sudden ventricular fibrillation at day 7 in the hospital. Continued treatment for 2 weeks ameliorated symptoms sufficiently to perform right cardiac catheterization and MRA of the heart, and the results showed a LVEF of 10%, mean PAP of 39 mmHg, and a PVR of 2.2 woods. The patient underwent heart transplantation after a combined treatment for 32 days.

The primary diagnosis of patient #3 was mirror dextrocardia (I.L.I.), situs inversus totalis, single atrium, common atrioventricular canal (type C), common atrioventricular valve insufficiency, pulmonary atresia, and patent ductus arteriosus. The patient underwent a bidirectional Glenn shunt at 2 years old and a total cavopulmonary connection at 4 years old. The patient had marked cyanosis of his lips, palpitations and dyspnea on exertion, and impaired exercise tolerance 10 years after TCPC. At that time, he had severe cyanosis of his extremities and face, frequent palpitations and dyspnea, and was sleeping in a semireclining position the month before hospitalization. The echocardiogram showed severe common atrioventricular valve insufficiency, and arterial blood gas analysis showed an arterial partial pressure of oxygen of 36.7 mmHg.

The primary diagnosis of patient #4 was dextroversion (A.L.L.), horizontal liver, asplenia, situs ambiguous, right atrial isomerism, bilateral superior vena cava, double-outlet right ventricle, common atrioventricular canal, functional single ventricle, severe common atrioventricular valve insufficiency, and pulmonary stenosis. The patient underwent a bilateral bidirectional Glenn shunt at 6 years old. The patient had marked palpitations, chest tightness, marked cyanosis of her extremities and face, edema of the lower limbs, and impaired exercise tolerance. These symptoms could not be improved after cardiotonic (digoxin) and diuretic treatment 1 year preceding the surgery. The patient had severe cyanosis of her extremities and face, frequent palpitations, dyspnea, ascites, and edema before hospitalization. The echocardiogram showed severe common atrioventricular valve insufficiency and a LVEF of 62%. Right cardiac catheterization showed that the mean PAP was 23 mmHg.

The primary diagnosis of patient #5 was mirror dextrocardia (I.L.I.), situs inversus totalis, single ventricle (type B), single atrium, and pulmonary stenosis. The patient underwent a bidirectional Glenn shunt at 3 years old and a total cavopulmonary connection at 13 years old. The patient had marked shortness of breath, abdominal distension, edema of the lower limbs, and impaired exercise tolerance. These symptoms improved slightly after cardiotonic and diuretic treatment 2 years before hospitalization, but clinical symptoms grossly worsened and could not be improved later. The patient had ascites, anorexia, edema, and vomiting before hospitalization. The echocardiogram showed severely diffuse weakening of ventricular wall motion and a LVEF of 19%, and right cardiac catheterization showed that the mean PAP was 12 mmHg.

### 3.3. Operative Technique

**Figure 1:**
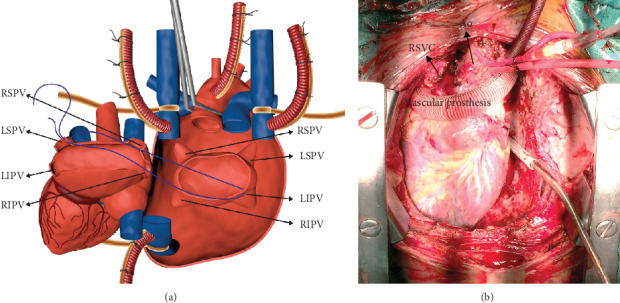
Outline of operative procedures of patients with cardiac dextroversion. (a) After the recipient cardiectomy, a standard left atrial cuff and a long cuff of systemic atrial tissue in continuity with the inferior vena cava was left. The atrial-atrial anastomosis was performed first between the left superior pulmonary vein (LSPV) orifice on the donor side and the left inferior pulmonary vein (LIPV) orifice on the recipient side. The heart was rotated rightward approximately 90° along its long axis and approximately 30° in the frontal plane. (b) The end-to-end anastomosis between the donor and recipient aorta was next. The recipient's IVC with an atrial cuff was used to create a large end-to-end anastomosis at the donor's IVC after enlarging the donor's IVC orifice. Patient #2 and #4's left-sided superior vena cava (LSVC) were connected with the donor's right atrial appendage by vascular prosthesis. Then, a large patch of the donor pulmonary artery was used to create a long anastomosis at the main pulmonary artery. Due to the rotation of the heart, the pulmonary artery was transposed to the right side of the aorta.

For three recipients (#1, #2, and #4) with dextroversion, right-sided or bilateral IVC, and right-sided SVC, and the aorta was cannulated for cardiopulmonary bypass. Recipient cardiectomy was performed, leaving a standard left atrial cuff and a long cuff of systemic atrial tissue in continuity with the inferior vena cava. The atrial-atrial anastomosis was performed first between the left superior pulmonary vein (LSPV) orifice on the donor side and the left inferior pulmonary vein (LIPV) orifice on the recipient side. The apex thereby rotated approximately 90° and to the right. The end-to-end anastomosis between the donor and recipient aorta was next. The recipient's IVC with an atrial cuff was used to create a large end-to-end anastomosis at the donor's IVC after enlarging the donor's IVC orifice. The end-to-end anastomosis between the donor's and recipient's right-sided SVC was next, and the recipient's left-sided SVC was connected with the right atrial appendage by vascular prosthesis above the reconstructed aorta for patients #2 and #4 with a bilateral SVC. A large patch of the donor pulmonary artery was used to create a long anastomosis at the main pulmonary artery. Because of the rotation of the heart, the pulmonary artery was transposed to the right side of the aorta (Figure 1).

**Figure 2:**
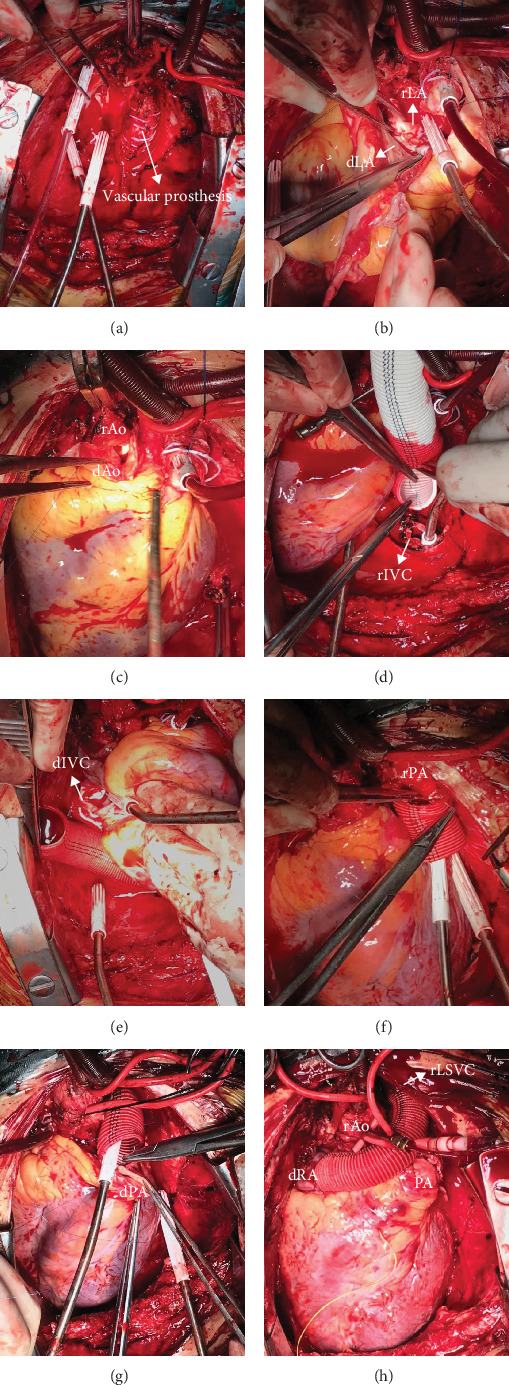
Outline of operative procedures of patients with mirror-image dextrocardia. (a) Left-sided IVC, femoral vein, and the aorta were cannulated for cardiopulmonary bypass. Recipient cardiectomy was performed, leaving a standard left atrial cuff. The vascular prosthesis of the last Fontan surgery was seen intraoperatively before removal. (b) The atrial-atrial anastomosis was performed first between the donor's left superior pulmonary vein (dLSPV) orifice and the recipient's left inferior pulmonary vein (rLIPV) orifice. (c) The anastomosis between the donor's aorta (dAo) and the recipient's aorta (rAo) was performed next. (d) The recipient's IVC (rIVC) was extended to the right by vascular prosthesis. (e) Vascular prosthesis anastomosed to the donor's IVC (dIVC) in an end-to-end fashion. (f) Owing to the lack of enough pulmonic tissue, the recipient's left pulmonary artery (rPA) was anastomosed to the vascular prosthesis. (g) The vascular prosthesis was anastomosed to the donor's pulmonary artery (dPA). (h) The orifice of the donor's SVC (dSVC) was closed, and the recipient's left-sided SVC (rLSVC) was connected with the right atrial appendage (rRA) by vascular prosthesis above the reconstructed aorta.

For the recipients (#3 and #5) with mirror-image dextrocardia and situs inversus totalis, left-sided SVC, femoral vein, and the aorta were cannulated for cardiopulmonary bypass. The atrial-atrial and aortic-aortic anastomoses were similar to those in the recipients with dextroversion. The recipient's IVC was extended to the right by vascular prosthesis, owing to an insufficient cuff of systemic atrial tissue, and then was anastomosed to the donor's IVC in an end-to-end fashion. Next, pulmonic anastomosis was performed. The donor's main pulmonary artery was connected with the recipient's left pulmonary artery by vascular prosthesis in an end-to-side fashion, owing to the lack of enough pulmonic tissue. Finally, the orifice of the donor's SVC was closed, and the recipient's SVC was connected with the right atrial appendage by vascular prosthesis above the reconstructed aorta (Figure 2).

### 3.4. Postoperative Condition and Follow-Up

The mean CPB (cardiopulmonary bypass) time and aortic clamping time were 190.2 ± 43.8 min and 65.8 ± 24.9 min, respectively. The mean ventilation time, ICU time, inotropic support time, hospitalization time, and 3-week postoperative EF were 59.6 ± 61.6 hours, 19.2 ± 22.3 days, 116.4 ± 69.9 hours, 41.6 ± 12.3 days, and 61.6 ± 1.5%, respectively (Table 3).

**Figure 3:**
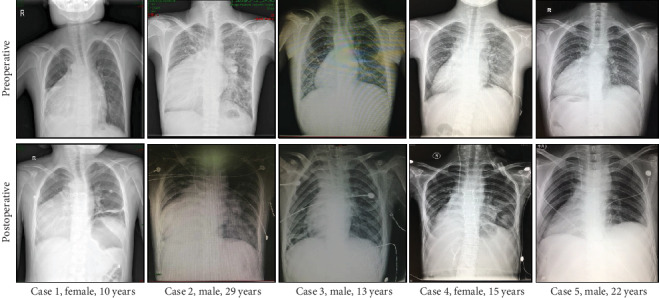
Preoperative and postoperative chest X-rays.

Cardiac echocardiography showed no obstruction of the vena cava in any patient 3 weeks after operation. All morphology, structure, and valve activity were good, and the systolic function of the dextrocardiac was normal 3 weeks after operation (Table 3). Postoperative chest X-rays of all patients showed that the heart was located on the right side, as shown in Figure 3.

Patients #1 and #4 were discharged from the hospital at days 26 and 35 after operation, respectively. Patient #2 was discharged at 43 days postoperation following the resolution of pneumonia. Patient #5 had delayed wound healing and was discharged from the hospital 45 days after operation, following wound healing. Patient #3 had severe pneumonia and hypoxemia. Percutaneous tracheotomy was performed 35 days after operation, and continuous ventilator-assisted breathing was performed. However, the control of pulmonary infection and oxygen saturation was poor. At 59 days after operation, the patient died of pulmonary infection and multiple-organ failure.

**Figure 4:**
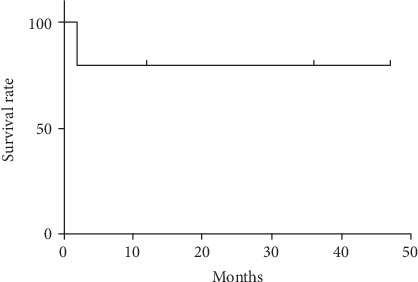
Survival analysis of the patients with CHD with dextrocardia after heart transplantation.

Patients #1, #2, #4, and #5 were followed up for 47 months, 36 months, 12 months, and 12 months, respectively. The mean follow-up time was 26.8 ± 17.6 months. The overall survival of the 5 patients at 1 month, 1 year, and 4 years was 100.0%, 80.0%, and 80.0%, respectively (Figure 4). The four surviving patients were back to NYHA class I and normal systolic function (LVEF > 55%) and did not have any clinical manifestations of rejection based on serum myocardial enzymes, echocardiography, and electrocardiography during the follow-up.

## 4. Discussion

Over the past 60 years, the diagnosis and treatment of congenital heart disease has made remarkable progress. Open-heart surgery and interventional treatment of structural heart disease have greatly improved the survival rate of patients with congenital heart disease. However, in the presence of extremely complex congenital heart disease, only 56% of newborns live to 18 years old. Successful surgical repair or palliative surgery can enable patients with complex congenital heart disease to reach adulthood. However, this treatment has never actually cured congenital heart disease, myocardial dysfunction, valvular heart disease, residual pulmonary hypertension, heart failure, or arrhythmia, all of which can lead to sustained morbidity and mortality [3]. Therefore, heart transplantation is the treatment for patients whose complex congenital heart is difficult to correct. Heart transplantation for CHD has continued to evolve since the initial case report in 1974 [6]. However, in spite of an increase in heart transplantation procedures over the last two decades, the percentage of heart transplantations that are performed for CHD patients remains low, especially in adults [2].

The surgical challenges and risks in heart transplantation for many forms of complex CHD are vastly different from those encountered in usual heart transplantation for cardiomyopathy. Dextrocardial CHD is a rare and special kind of complex CHD in which the apex and long axis of the heart are located on the right side; thus, matching a donor with dextrocardia is virtually impossible. Therefore, a normal-position donor heart is transplanted to patients with dextrocardial CHD. This procedure demands great technical challenge and entails great risk. The main reasons include (1) the abnormal position of the dextrocardiac makes it difficult to place the left donor heart during transplantation; (2) there are often complex structural anomalies, as listed in Table 2, for the patients in our institute; (3) the dextrocardiac is often accompanied by an abnormal distribution of the great vessels and complex arteriovenous connection; (4) some patients have had previous palliative procedures leading to severe adhesions; and (5) some patients may be accompanied by pulmonary vascular dysplasia, increased aorta-pulmonary artery collateral vessels, and increased pulmonary vascular resistance.

In response to these difficulties and challenges, some heart centers have reported some solutions. Dextrocardia includes dextroversion (situs solitus or ambiguous) and mirror-image dextrocardia (situs inversus totalis). The dextroversion with situs solitus or situs ambiguous is right-sided or bilateral SVC and right-sided IVC, while mirror-image dextrocardia with situs inversus totalis is left-sided SVC and left-sided IVC. The first heart transplantation for CHD was reported in 1974 [6]. Initial heart transplantation strategies inserted the donor heart into the left thoracic cavity by incision of the left pericardium for patients with dextroversion. In 1990, Doty et al. [7] first reported an approach for heart transplantation in patients with dextrocardia in situs inversus totalis. They used extracardiac reconstruction of the systemic venous return to the heart after total excision of the right atrium. They reconstructed the superior vena cava using the innominate vein on the right side of the recipient and the superior vena cava of the donor. Rerouting of the inferior vena cava required the construction of a composite conduit using the right atrium and the in situ pericardium over the diaphragm. In 1995, Michler and Sandhu [8] created two autologous left-sided atrial tissue baffles tunneling the left superior vena cava and inferior vena cava to the right of the pulmonary veins. Rubay et al. [9] handled abnormal lesions of the vena cava by aortic homograft of the recipient's right atrial wall and pericardium. However, the above approaches maintained levocardia and could not avoid pericardial resection, lung compression, and right ventricular compromise [10], which could be further proved by a cohort study. In 2008, Deuse and Reitz [11] reported a case of heart transplantation while maintaining dextrocardia in situs inversus totalis. The donor graft was then prepared by oversewing the left pulmonary vein orifices and opening the left atrium between the right pulmonary veins. The pulmonary atrial anastomosis was performed first between the opening of the right pulmonary vein orifices on the donor side and the atrial cuff on the recipient side. The heart was rotated rightward approximately 45° along its long axis and approximately 30° in the frontal plane. The apex thereby rotated approximately 120° to the right. The results reported by Reinhartz et al. [10] at the same institute showed that this technique maintained excellent long-term outcomes at the ten-year follow-up. It avoided pericardial resection, lung compression, and right ventricular compromise. Anastomotic obstruction of the SVC connection to the right atrium may occur approximately 20% of the time and is amenable to transcutaneous stenting.

For heart transplantation with dextrocardial CHD, our experience dictated that a long enough aorta, pulmonary artery, and superior and inferior vena cava be retained when the donor heart was harvested, and innominate veins were retained when necessary. Then, a standard left atrial cuff and a long cuff of systemic atrial tissue in continuity with the inferior vena cava were left when recipient cardiectomy was performed. The atrial-atrial anastomosis was first performed between the left-upper pulmonary vein orifice on the donor side and the left-lower pulmonary vein orifice on the recipient side. The apex thereby rotated approximately 90° to the right. The end-to-end anastomosis between the donor and recipient aorta followed. For heart transplantation with dextroversion, the above simple technique could maintain dextrocardia after heart transplantation and avoid pericardial resection, lung compression, and right ventricular compromise. For children younger than 18 years old, vascular prosthesis was always avoided for its long-term low flow velocity. The third patient had two previous palliative procedures (a Glenn procedure and a TCPC procedure) in addition to the above technique. The recipient's IVC was extended to the right by a Dacron vascular graft, owing to an insufficient cuff of systemic atrial tissue, and then was anastomosed to the donor's IVC in an end-to-end fashion. The orifice of the donor's SVC was closed, and the recipient's SVC was connected with a right atrial appendage by a Dacron vascular graft before reconstructing the aorta. Therefore, the vascular prosthesis and the autologous or xenogenous pericardium were used to reconstruct the mirror-image systemic and pulmonary venous pathways for patients with a previous palliative procedure.

For patients with the Fontan failure undergoing heart transplantation, the mortality rate was higher than for patients with the non-Fontan CHD and non-CHD [12]. The Fontan circulation is different than normal circulation. The Fontan neoportal system dams off and pools the systemic venous blood. As a result, the transit of blood through this neoportal system depends on the pressure gradient from the systemic postcapillary vessels to the pulmonary postcapillary vessels. As there is no pump to add energy to the system, small changes in the static resistances and dynamic impedances of the structures within this portal system have a profound impact on the blood flow. This Fontan failure is complicated by pulmonary arteriovenous fistula, increasing PVR, protein-losing enteropathy (PLE), plastic bronchitis, or liver fibrosis/cirrhosis, each of which markedly increases the mortality rate [13]. It has been reported that the 1-year survival rate is approximately 70% [12]. In case 3, the circulation of the patient was stable, and persistent hypoxia prevented the patient from leaving ventilator assistance. Ultimately, infection resulted in multiorgan failure and death. With widespread use of bidirectional cavopulmonary anastomosis or the Fontan procedure as palliation for children with single-ventricle physiology, pulmonary arteriovenous fistulas (PAVFs) continue to be causes of considerable morbidity [14]. The PAVFs after a cavopulmonary anastomosis can be attributed mainly to hepatic factors [15].

We speculate that hypoxemia caused by extensive diffuse PAVFs acted as a cause of death for patient #3, but preoperative pulmonary angiography did not indicate obvious PAVFs or aortopulmonary collateral vessels. Therefore, accurately evaluating PAVFs is a critical and difficult problem. For patients with the Fontan failure who mainly manifest with progressive hypoxemia, physicians should be aware of the possibility of extensive diffuse PAVFs. There are insufficient clinical data to determine whether patients with the Fontan failure should undergo heart transplantation or heart-lung combined transplantation. The Fontan failure with preserved systolic ventricular function (PVF) is an independent risk factor for mortality after heart transplantation. The five-year survival rate for Fontan-failure patients with PVF receiving heart transplantation is 40-60%, compared with an 80-89% five-year survival rate of heart transplantation patients with impaired systolic function [16].

## 5. Conclusions

Heart transplantation with dextrocardial CHD is rare. Rotation of the left atrium by 90°, aortic end-to-end anastomosis and vena caval reconstruction by vascular prosthesis or systemic atrial cuff is a simple and effective surgical technology. The short-term and medium-term follow-up showed good results, especially for patients with dextroversion, but the long-term results need further follow-up.

## Figures and Tables

**Table 1:** Preoperative characteristics among the patients with CHD with dextrocardia.

		No. 1	No. 2	No. 3	No. 4	No. 5
Recipient	Sex	Female	Male	Male	Female	Male
	Age (years)	11	29	13	15	22
	Weight (kg)	26	44	49	35	75
	LVEF (%)	21	28	39	62	19
	NYHA class	IV	III	IV	IV	IV
	BNP (pg/ml)	3982	3982	3982	4000	2670
	Mean PAP (mmHg)	30	30	30	23	12
	ABO type	B	B	B	B	O
Donor	Gender	Male	Male	Male	Female	Male
	Age (years)	40	46	19	13	38
	Weight (kg)	65	60	55	40	50
	ABO type	B	B	B	O	O
	Cause of brain death	Cerebrovascular accident	Cerebral tumor	Cerebral trauma	Cerebrovascular accident	Cerebral trauma
	Cold ischemic time (min)	130	248	375	400	270

CHD: congenital heart disease; LVEF: left ventricular ejection fraction; NYHA: New York Heart Association functional classification; BNP: brain natriuretic peptide.

**Table 2:** Diagnosis of the patients with CHD with dextrocardia.

	Diagnosis	Past cardiac surgery
No. 1	Common atrioventricular canal, single atrium, single ventricle, severe common atrioventricular valve insufficiency, and pulmonary stenosis	
No. 2	Double-outlet right ventricle, bilateral superior vena cava, ventricular septal defect, pulmonary hypertension, and arrhythmia	
No. 3	Pulmonary atresia, single atrium, common atrioventricular canal, and severe common atrioventricular valve insufficiency	Glenn, age of 1 TCPC, age of 3
No. 4	Right atrial isomerism, double-outlet right ventricle, common atrioventricular canal, functional single ventricle, severe common atrioventricular valve insufficiency, and pulmonary stenosis	Glenn, age of 6
No. 5	Single ventricle (type B), single atrium, and pulmonary stenosis	Glenn, age of 4 TCPC, age of 12

CHD: congenital heart disease; TCPC: total cavopulmonary connection.

**Table 3:** Postoperative characteristics and survival status among the patients with CHD with dextrocardia.

	No. 1	No. 2	No. 3	No. 4	No. 5
CPB time (min)	174	127	238	188	224
Aorta clamp time (min)	105	38	53	70	63
Ventilation time (min)	19	24	168	44	43
ICU stay time (d)	9	8	59	12	8
Inotropic support time (h)	38	48	136	192	168
Hospitalization time (d)	26	43	59	35	45
LVEF 3 postoperative week (%)	63	59	61	60	60
Complications	No	Pneumonia	Pneumonia, hypoxemia	No	Delayed wound healing
Survival status	Yes	Yes	No	Yes	Yes

CHD: congenital heart disease; CPB: cardiopulmonary bypass; ICU: intensive care unit; LVEF: left ventricular ejection fraction.

## Data Availability

(1) The (surgery procedure, postoperative X-rays, and perioperative and postoperative patients' characteristics) data used to support the findings of this study are included within the article. (2) Other (perioperative treatment of the heart transplant patients) data used to support the findings of this study are available from the corresponding author upon request.

## Abbreviations

ISHLT: The International Society for Heart and Lung Transplantation
CHD: Congenital heart disease
CAVC: Common atrioventricular canal
TCPC: Total cavopulmonary connection
PVO: Pulmonary vein orifices
SVC: Superior vena cava
IVC: Inferior vena cava
PAP: Pulmonary artery pressure
DBD: Donation after brain death
LSPV: Left superior pulmonary vein
LIPV: Left inferior pulmonary vein
PVR: Pulmonary vascular resistance
PLE: Protein-losing enteropathy
PAVF: Pulmonary arteriovenous fistulas
PVF: Preserved systolic ventricular function
